# Successful Treatment With Omalizumab in a Child With Asthma and Urticaria: A Clinical Case Report

**DOI:** 10.3389/fped.2019.00213

**Published:** 2019-06-05

**Authors:** Maria Maddalena Sirufo, Lia Ginaldi, Massimo De Martinis

**Affiliations:** ^1^Department of Life, Health and Environmental Sciences, University of L'Aquila, L'Aquila, Italy; ^2^Allergy and Clinical Immunology Unit, Department of Medicine, AUSL 04, Teramo, Italy

**Keywords:** chronic spontaneous urticaria, asthma, child, omalizumab, hypersensitivity, translational medical research, precision medicine, allergy

## Abstract

Childhood urticaria is not rare, although its persistence is less frequent. In children, chronic spontaneous urticaria (CSU) is associated with comorbidities, including asthma, allergic rhinitis, or atopic dermatitis, and many children with CSU have a family history of atopy. The therapeutic approach to CSU in children is the same one recommended by international guidelines for treatment of chronic urticaria in adults. In the European Union, according to the European Medicine Agency, omalizumab is the add-on drug of choice for the management of CSU in adult and adolescent patients (from 12 years of age) with inadequate response to H1 antihistamine therapy. In addition, in children (6 to <12 years of age), it is the add-on therapy of choice to improve asthma control. The management of children with urticaria under 12 is a therapeutic area with few certainties, where omalizumab can be administered only “off-label.”

## Background

Chronic urticaria (CU) is a common skin disorder, characterized by the appearance of pruritic wheals or angioedema (and—in about 40% of cases—both manifestations), persisting for longer than 6 weeks. Due to its frequent and persevering itching attacks, it significantly impacts the quality of life of patients and their caregivers, with significant implications on their physical and psychological state; in children, it has been demonstrated that this condition is associated with a reduced school performance (1). CU can be differentiated into spontaneous or inducible urticaria, based on the trigger factor. Inducible urticaria is due to a particular stimulus, that can be mechanical (friction, pressure, vibration), thermal (hot, cold), or electromagnetic (solar radiation). It occurs after a short interval from the onset of the stimulus and regresses following the removal of the same. On the contrary, in chronic spontaneous urticaria (CSU), wheals are often associated with angioedema, are not predictable, and last from a few days to weeks (2). CSU originates from mast-cell activation, which releases histamine and other mediators (leukotrienes, prostaglandins, and platelet-activating factor) capable of inducing sensory nerve activation, vasodilatation, and plasma extravasation (3). Immediate hypersensitivity (type I) is the most common immunological disease and represents the most widely spread and the fastest-growing chronic human health condition in people from 15 years of age (4).

Epidemiological data in children are scarce. Childhood urticaria is not rare (3.4–5.4%), although its persistence is less frequent (0.1–0.3%). According to recent data from Korea, the prevalence of CU is 1.8%. The trigger (or cause) of CU in children can be identified in 21–55% of cases. It can be associated with infections (viral, bacterial, parasitic, or helminthic), drugs, food or food additives, thyroid disease, or physical stimuli, but it is often an autoreactive/autoimmune/autoallergic process and, unlike in adults, identifying auto-antibodies in children does not affect the disease prognosis (1, 5).

In children, CSU is associated with comorbidities, 28.1% of which are represented by atopy (asthma, allergic rhinitis, or atopic dermatitis); in fact, 43.0% of children with CSU have a family history of atopy (6).

Among these comorbidities, asthma is the most common chronic lung disease in pediatric patients, and in 2–5% of cases, it remains symptomatic, despite the implementation of addressing modifiable risk factors and an adequate therapy (high-dose inhaled corticosteroids, long-acting β2-receptor agonists, leukotriene receptor antagonists, and occasionally, systemic steroids) (7).

According to a consensus-based recommendation, the therapeutic approach to CSU in children should include the use—with caution—of the same algorithm of adults (1). The first-line treatment in the pediatric population is based on second-generation, non-sedating antihistamines; only medications with proven safety and efficacy should be administered, such as cetirizine, desloratadine, fexofenadine, levocetirizine, rupatadine, bilastine, and loratadine. All further steps should be taken carefully, and based on individual considerations, since up-dosing of antihistamines and further treatment options (omalizumab, ciclosporin) have not been studied adequately in children.

Omalizumab is an immunoglobulin E specific IgG1 k antibody (recombinant DNA-derived humanized monoclonal antibody) that targets circulating free IgE. Omalizumab binds to IgE and lowers free IgE levels; consequently, IgE receptors (FcεRI) on cells down-regulate. How this produces an improvement of CSU symptoms is not entirely understood (8).

In the European Union—according to the European Medicine Agency—omalizumab in children (6 to <12 years of age) is indicated as add-on therapy to improve asthma control in patients with severe persistent allergic asthma, who present a positive skin test or *in vitro* reactivity to a perennial aero-allergen and frequent daytime symptoms or night-time awakenings, and who have suffered from multiple documented severe asthma exacerbations, despite the daily use of high-dose inhaled corticosteroids, plus a long-acting inhaled beta2-agonist. Omalizumab is also indicated as add-on therapy for the treatment of CSU in adult and adolescent patients (from 12 years of age) with inadequate response to H1 antihistamine treatment.

Guidelines recommend the administration of omalizumab as a subcutaneous injection; the dosing and frequency of administration in asthma are guided by a nomogram derived from the total serum immunoglobulin E level and the body mass index, while in CU, a fixed 300-mg dose is administered every 4 weeks.

The treatment of children with urticaria under 12 is a therapeutic “limbo,” where omalizumab can be administered only “off-label.”

## Case Report

We report the case of an 8-year-old child (weight, 38 kg; height, 137 cm) with severe asthma sensitized to *Dermatophagoides pteronyssinus* and *D. farinae*. At age 6, he was started on budesonide/formoterol fumarate dihydrate 80 μg/4.5 μg (two inhalations twice a day), montelukast 5 mg once a day, and cetirizine 5 mg once a day, without any long-term or satisfactory relief from the symptoms. In addition, over the last year, despite a treatment with antihistamine and antileukotriene, there was no satisfactory improvement. The child also presented CU, which temporarily regressed with betamethasone 4 mg a day, discontinued due to poor tolerance and induction of psychomotor agitation.

A complete medical history was obtained and a physical examination was performed at the time of diagnosis and upon each subsequent revaluation.

The physical examination showed evanescent coalescing wheals on the trunk and limbs.

At our first spirometric evaluation, the child had a FEV_1_ < 65%. The diagnosis of CSU is based on EAACI/GA2LEN/ EDF/WAO guidelines, and the evaluation of the clinical efficacy of omalizumab during therapy has been monitored with the Urticaria Activity Score (UAS 7) (2), which, before treatment, was 35.

Laboratory investigations—including complete blood count with differential, standard biochemical profile, erythrocyte sedimentation rate, C-reactive protein, serum protein electrophoresis, d-dimer, thyroid function screening, serum protein electrophoresis, vitamin D, anti-nuclear antibodies, and stool examination for parasites—were performed, with negative results. Also negative was the autologous serum skin test performed. Total IgE was 82.8 kUA/l and serum-specific IgE positive to house dust mites was 6.63 KUA/l (*D. pteronyssinus*) and 4.03 KUA/l (*D. farinae*). Serum IgE reactivity was analyzed using the ISAC platform (Thermo Fisher Scientific, Uppsala, Sweden), by means of the latest commercially available ImmunoCAP-ISAC, as per manufacturer's instructions.

Instrumental examinations (chest X-ray, electrocardiogram, abdominal ultrasound scan, and urea breath test) did not reveal any significant pathological finding.

Omalizumab is now recommended—and widely used—as a first-line add-on treatment option in severe allergic asthma in children resistant to treatment (Global Initiative for Asthma—GINA), and is highly effective and safe (9).

The need for a more adequate asthma treatment associated with failed response to conventional treatment for CSU and intolerance to systemic corticosteroids in our patient and the good tolerance of omalizumab in asthmatic children older than 6 were the reasons for our choice of this drug. Therefore, an anti-IgE treatment was proposed to the child's parents and—after obtaining their informed consent, in compliance with the rules of our Institution—omalizumab was administered at room temperature (75 mg via subcutaneous injection per month). The dose of omalizumab we administered to our patient was that recommended in the treatment of asthma in children.

Cetirizine, montelukast, and budesonide/formoterol fumarate were gradually tapered down and stopped at week 24 of the omalizumab therapy. Table 1 reports the values of UAS7 and FEV_1_ for the observation period.

**Table 1:** Clinical and therapeutic assessment of patient throughout omalizumab treatment.

**Month**	UAS 7^*^	**FEV 1% Predicted**	**FVC**	**FEV1/FVC**	**Total IgE**	**Specific IgE**	**WBC**	**Neutrophils**	**Basophils**	**Eosinophils**	**Cetirizine**	**Montelukast**	**Budesonide/formetherolfumaratedihydrate 80 μg/4.5 μg**	**Omalizumab**
0	35	65	5.26	−0.9	82,800 KUA/l	*Dermatophagoides pteronyssinus* 6.63 KUA/l*Dermatophagoides farinae* 4.03 KUA/l	7.60 × 10^3^/μL	48.1%−3.65 × 10^3^/μL	0.08%−0.06 × 10^3^/μL	5.3%−0.40 × 10^3^/μL	5 mg	5 mg	Two inhalations twice a day	75 mg
1	29										5 mg	5 mg	Two inhalations twice a day	75 mg
2	20										5mg	5 mg	Two inhalations twice a day	75 mg
3	14										5 mg	5 mg	Two inhalations twice a day	75 mg
4	7										0	0	Two inhalations twice a day	75 mg
5	0										0	0	Two inhalations once a day	75 mg
6	0	85	4.20	1.2							0	0	0	75 mg
9	0										0	0	0	75 mg
12	0	95	5.30	5.9	369 KUA/l	*Dermatophagoides pteronyssinus >*100 KUA/l*Dermatophagoides farinae* 81.5 KUA/l	10.83 × 10^3^/μL	53.7%−5.82 × 10^3^/μL	0.7%−0.07 × 10^3^/μL	4.1%−0.44 × 10^3^/μL	0	0	0	75 mg
18	0	95	5.25	5.6							0	0	0	75 mg

* *7-day urticaria activity score (UAS7)*.

After 3 months of therapy, UAS7 was reduced by 50% (UAS 14), and after 5 months, it was reduced by 100% (UAS 0). After 1 year, the remission of urticaria persists, confirming the resolution of the disease.

After 6 months of therapy, the patient showed an improvement in his asthmatic symptoms, in agreement with the global spirometry values (FEV_1_95%).

Moreover, the patient did not manifest any adverse effects due to the omalizumab therapy. This work has been conducted in compliance with the relevant ethical standards and the norms established by the Internal Review Board of the University of L'Aquila (*Comitato etico di Ateneo* D.R.n.206/2013 and D.R.n.46/2017).

The patient's parents provided their written informed consent for the publication of this report.

## Discussion

We have described the comorbidity of asthma and CSU and its treatment with omalizumab in an 8-year-old boy. CSU has a complex and multiform pathogenesis; therefore, its diagnosis and management, especially in children, is still uncertain.

Being clinically effective and safe, omalizumab now represents an obvious alternative treatment in subjects that are refractory to the antihistamine therapy. In adults treated with omalizumab, hives, itching, and angioedema are significantly reduced, while the patient's quality of life improves (10, 11).

In children, as in adults, the treatment of urticaria is essentially the same, with a preference for second-generation over first-generation antihistamine, in order to avoid the sedating effects typical of the latter. Corticosteroids should be avoided, due to their growth-related side effects, or at least they should be used for short periods <10–14 days (12, 13).

Ben-Shoshan and Grattan reviewed the diagnosis and management of CSU in children. Their findings supported the use of omalizumab for the most severe cases, while highlighting the effectiveness of second-generation antihistamines for the treatment of CSU (14).

Omalizumab—an anti-IgE antibody—seems to be promising in a wide range of allergic diseases, and so far has shown a robust clinical efficacy in the treatment of moderate to severe persistent asthma and chronic idiopathic urticaria in adults (15, 16). Its primary action is neutralizing free serum IgE, thus reducing the amount that would normally be available to bind to FcεRI on mast cells and basophils and trigger the allergic cascade. Furthermore, in atopic subjects, treatment with omalizumab produces a marked down-regulation of FcεRI receptors on basophils.

Indeed, the specific mechanism of action of omalizumab in the various diseases in which it has proven effective is still partly unknown. Several mediators orchestrate the allergic response, and omalizumab showed to act on both the early and the late phases of the allergic reaction, with a reduction of the proinflammatory cytokine levels and dendritic cells, lymphocyte, and eosinophil counts in tissues, eventually decreasing the inflammatory state of the whole immune system.

Comorbidity is quite frequent in the atopic disorders in children (17) but, in this case and in our opinion, the coexistence of both conditions at that age in relation to treatment is of particular significance. In fact, omalizumab is approved for the treatment of asthma in children from the age of 6 and for the treatment of CSU from the age of 12, and the posology of the drug is different in the treatment of the two diseases.

The need for a treatment with omalizumab in children under the age of 12 is rare, and few cases of children with CSU under 12 successfully treated with omalizumab are described in the literature (18, 19). The satisfactory response to omalizumab in our child, after the failure of previous standard therapeutic strategies, seems to confirm the effectiveness and the safety of this molecule in the treatment of urticaria as well as asthma, as already highlighted in other studies in adults (8). We also show that, in children with CU, omalizumab could be effective at a lower dose than in adults.

Another relevant learning of this case is the fact that it could be possible to prescribe the optimal treatment to the right patient, tailoring the most appropriate therapy to the individual patient, as suggested in children by recent guidelines (2). Precision medicine—that is, the choice of a personalized treatment based on the exact characteristics of the disease and the single patient—should be a requirement, especially in pediatrics.

In conclusion, controlled studies are needed on the use of omalizumab in children with CSU younger than 12. However, based on literature data and on our experience, it seems that an anti-IgE therapy could be a safe and effective alternative for the treatment of these young patients who have not responded to first-line treatment, possibly using a personalized approach.

## Ethics Statement

The case described in compliance with the rules of our Institution not necessitate the approval of ethics committee.

## Author Contributions

All authors listed have made a substantial, direct and intellectual contribution to the work, and approved it for publication.

### Conflict of Interest Statement

The authors declare that the research was conducted in the absence of any commercial or financial relationships that could be construed as a potential conflict of interest.
